# Acceptability of a Community-Based Outreach HIV-Testing Intervention Using Oral Fluid Collection Devices and Web-Based HIV Test Result Collection Among Sub-Saharan African Migrants: A Mixed-Method Study

**DOI:** 10.2196/publichealth.5519

**Published:** 2016-08-04

**Authors:** Jasna Loos, Lazare Manirankunda, Tom Platteau, Laura Albers, Katrien Fransen, Tine Vermoesen, Fiona Namanya, Christiana Nöstlinger

**Affiliations:** ^1^ HIV and Sexual Health Unit Department of Public Health Institute of Tropical Medicine Antwerp Belgium; ^2^ Helpcenter-ITM Department of Clinical Sciences Institute of Tropical Medicine Antwerp Belgium; ^3^ AIDS Reference Laboratory Department of Clinical Sciences Institute of Tropical Medicine Antwerp Belgium

**Keywords:** oral fluid collection devices, Web-based HIV test result, sub-Saharan African migrants, outreach HIV testing, acceptability

## Abstract

**Background:**

Late human immunodeficiency virus (HIV) diagnosis is common among sub-Saharan African migrants. To address their barriers to HIV testing uptake and improve timely HIV diagnoses and linkage to care, the outreach HIV testing intervention, “swab2know,” was developed. It combined a community-based approach with innovative testing methods: oral fluid self-sampling and the choice between Web-based HIV test result collections using a secured website or post-test counseling at a sexual health clinic. The sessions included an informational speech delivered by a physician of sub-Saharan African origin and testimonies by community members living with HIV.

**Objectives:**

The objectives of this study were to evaluate the intervention’s acceptability among sub-Saharan African migrants and its potential to reach subgroups at higher risk for HIV infection and to identify facilitators and barriers for HIV testing uptake.

**Methods:**

This mixed-method study combined qualitative (participant observations and informal interviews with testers and nontesters) and quantitative data (paper–pencil survey, laboratory data, and result collection files). Data were analyzed using a content analytical approach for qualitative and univariate analysis for quantitative data.

**Results:**

A total of 10 testing sessions were organized in sub-Saharan African migrant community venues in the city of Antwerp, Belgium, between December 2012 and June 2013. Overall, 18.2% of all people present (N=780) underwent HIV testing; 29.8% of them tested for HIV for the first time, 22.3% did not have a general practitioner, and 21.5% reported 2 or more sexual partners (last 3 months). Overall, 56.3% of participants chose to collect their HIV test results via the protected website. In total, 78.9% collected their results. The qualitative analysis of 137 participant observation field notes showed that personal needs and Internet literacy determined the choice of result collection method. Generally, the oral fluid collection devices were well accepted mainly because sub-Saharan African migrants dislike blood taking. For some participants, the method raised concerns about HIV transmission via saliva. The combination of information sessions, testimonies, and oral fluid collection devices was perceived as effectively reducing thresholds to participation. Acceptability of the intervention differed between individual participants and settings. Acceptance was higher among women, in churches and settings where community leaders were engaged in HIV awareness raising. Higher preventive outcomes were observed in settings with lower acceptance. The presence of the intervention team visualized the magnitude of the HIV epidemic to the public and promoted HIV testing uptake at large, for example, those who declined indicated they would take up testing later.

**Conclusions:**

When accompanied by tailored provision of information, outreach HIV testing interventions adopting a community-based approach and innovative methods such as Web-based result collection and oral fluid collection devices are acceptable and reduce thresholds for HIV testing uptake. The swab2know intervention was able to reach sub-Saharan African migrants at risk of HIV infection, and with limited access to regular HIV testing. Among nontesters, the intervention contributed to awareness raising and therefore has a place in a multipronged HIV test promotion strategy.

## Introduction

The human immunodeficiency virus (HIV) epidemic among sub-Saharan African migrants in Europe reflects the generalized epidemics in sub-Saharan Africa. Next to men who have sex with men (MSM), HIV is concentrated in the small communities of sub-Saharan African migrants [1], constituting the second largest group affected by HIV in Belgium [2]. Most of them are believed to have acquired HIV heterosexually in their country of origin, but there is increasing evidence to show that post-migration HIV infection is occurring in Europe [3,4]. Structural factors related to migration [5,6], closely knitted sexual networks of sub-Saharan African migrants [7], and high proportions of late diagnoses [1,2] increase sub-Saharan African migrants’ risk for HIV infection in host countries. Greater investment in uptake of HIV testing and linkage to care can alter this trend. Early diagnoses reduce the individual morbidity and mortality of patients and health care costs. Although the advantages of HIV testing are well known by the sub-Saharan African migrant communities, they do not translate in regular uptake [8]. A body of literature describes the multiple and intertwined barriers at individual, community, and health care level for this key population [5,8-14]. To reduce thresholds for HIV testing uptake, interventions both in the communities and at health care level [15,16] have been set up in Flanders. Community-level interventions were developed using a participatory approach and adopted outreach and community mobilization strategies to reach the heterogeneous sub-Saharan African migrant communities [15]. Evidence from Africa suggests that outreach HIV testing interventions can be effective in accessing “hard-to-reach” populations [17,18] and lead to earlier diagnosis of HIV [19,20]. A first pilot project, offering free HIV tests in sub-Saharan African migrant and MSM community settings, yielded good primary prevention benefits, but groups with higher risk of undiagnosed HIV infection were not reached [21]. A follow-up intervention, labeled “swab2know,” aimed at further reducing thresholds for testing while preserving the preventive outcomes by adopting new, less invasive HIV-testing technologies. To overcome fears and barriers related to blood taking [22], oral fluid collection devices were introduced (Oracol device; Malvern Medical Developments, Worcester, UK). This method was previously validated for the purpose of screening among populations with HIV prevalence above 1%, indicating that it is suitable for sub-Saharan African migrants [23,24]. Result collection often has been described as low in mobile Voluntary HIV counseling and testing (VCT) interventions [21,25]. To reduce failures to return for HIV test results, the option to collect them using a secured website was given. In addition, result communication through traditional post-test counseling at a low-threshold HIV or sexually transmitted infections (STI) testing facility focused on groups with a high risk of acquiring HIV or STIs like sub-Saharan African migrants were also offered *.* In this context, low-threshold HIV or STI testing facility is defined as an outpatient testing site offering free and anonymous HIV testing for the main groups at risk for HIV/STIs, that is, MSM, sub-Saharan African migrants, and young people. The post-test counseling approach used was in line with the recommendations made by COBATEST, a European network on community-based voluntary counseling and testing centers, which has acquired extensive experience on HIV community–based testing practices in Europe and published guidance on this topic [26]. Both methodologies—oral fluid sample collection and Web-based test result communication—were novel to Belgium’s sub-Saharan African migrant communities. The detailed methodology of the “swab2know” intervention and first results pertaining to testing uptake at MSM community settings in Antwerp city have been described elsewhere [27].

To evaluate this intervention’s potential to detect new HIV infections among sub-Saharan African migrants and link them to care (eg, HIV test result collection and referral to HIV-specialized care for people with an HIV diagnosis), we conducted a mixed-method study in different community settings. We describe the key findings in terms of the interventions’ acceptability among sub-Saharan African migrants, as well as facilitators and barriers for intervention uptake.

## Methods

### Intervention Methods: “Swab2know” Methodology

Between December 2012 and June 2013, 10 “swab2know” outreach VCT sessions were organized in self-selected community settings of the largest sub-Saharan African migrant communities residing in the city of Antwerp, that is, the Demographic Republic of Congo (DRC), Nigeria, Ghana, and Cameroon. Community settings included churches, bars, events, and information sessions of African organizations. More specifically, we visited a Cameroonian, Nigerian, and Ghanaian organization, an African Lesbian Gay Bisexual Transsexuals (LGBT), an African women organization, and a Congolese youth group. The intervention adopted a settings approach, for example, all people present in the community settings, sub-Saharan African migrants as well as people from other origins were invited to participate, except minors. The rationale behind this approach was that not only sub-Saharan African migrants are at risk for HIV but also their sexual partners regardless of their origin or migration status.

The “swab2know” sessions included an introduction of the testing offer by a medical doctor of sub-Saharan African origin and a testimony of a community member living with HIV. Interested participants received detailed explanation of the study procedures in a separate area. Participants who accepted to participate were asked to read and sign an informed consent form and to fill a short questionnaire. They self-collected an oral fluid sample through an Oracol collection device. Participants preferring to receive their HIV test result at the HIV or STI testing facility immediately got an appointment. Those choosing the secured website [27] were assisted in creating a personal account through which they could access their results. They received automated email reminders when their results were available. Before the study, community leaders had recommended to not communicate test results indicating an HIV diagnosis via the website. Following this advice, participants were informed that in case of reactive or invalid samples, they would receive a mobile phone call from the study nurse. All data items were linked with a unique code to guarantee confidentiality in handling the data. Within 7 days of collecting the sample, an HIV ELISA (Genscreen HIV-1/2 version 2 BioRad, Marnes-la-Coquette, France) was performed at the AIDS Reference Laboratory of the Institute of Tropical Medicine, according to a validated protocol for oral fluid specimens. This test gives a 100% (95% CI: 95.9-100) sensitivity and 97.6% (95% CI: 94.5-99.0) specificity [28] in established HIV-untreated individuals. In addition, the quality of the oral fluid samples was measured using the IgG ELISA quantification kit (Human IgG ELISA Immunology Consultants Laboratory, Inc., Portland, OR, USA). If the sample contained more than 3500 ng total IgG/mL, it was considered valid.

Approximately 1 week after participation, HIV test results were made available to the participants. As recommended by the community leaders, participants with a reactive HIV result received a mobile phone call from the study nurse inviting them for counseling at the STI and HIV testing center. During the posttest counseling session, it was explained that results indicative for an HIV infection required confirmation by a traditional blood test, which was strongly advised. Participants whose results were confirmed were referred to the AIDS Reference Clinic of the Institute of Tropical Medicine. Participants with a nonreactive sample who chose to collect their result via the secured website found the message as shown in Textbox 1.

**Website message delivered to participants with a nonreactive HIV test result for Web-based test result collection (available in Dutch, French, and English).**

Your saliva has not reacted to the test. We can conclude with high reliability that you are not infected with HIV. Please note that an HIV test does not give any information about the risk you may have had during the last three months. Furthermore, an oral fluid test is not officially recognized to diagnose an HIV infection.

If you ran a sexual risk during the last three months it is better to have a combined HIV test performed on your blood (antibody and antigen determination). This will give a better (but not absolute) result for recent risks. After having had unsafe sexual contacts, it can be important to test for sexually transmitted infections. To discuss the necessity of such tests, you can contact your GP, an HIV specialized treatment center or Helpcenter. (www.helpcenteritg.be)

When you have casual sex with different partners, it is recommended to have an HIV test every 3-6 months.

We ‘d like to contact you by email or text message in 3-6 months, so you can order a test kit for a new oral fluid sample.

If you don’t want to receive this email, please let us know by changing the settings of your account.

If we send a test to you, you can collect the sample yourself and send it to ITM’s laboratory using the envelope provided. The procedure for obtaining your result will be the same.

In case participants missed the initial appointment or failed to pick up the results from the website, the study nurse made up to 4 attempts to contact participants and encourage them to collect their results to ensure linkage to care. In case of reactive test results, up to 8 contact attempts were made.

### Research Methods

This prospective interventional study used mixed methods to answer the research questions, capitalizing on each method’s strengths to obtain a nuanced understanding of the intervention’s potential to detect new HIV infections and to link participants to care.

### Quantitative Methods

To identify participants’ characteristics, including their risk for an undiagnosed HIV infection, a brief self-reported 11-item questionnaire was used to assess sociodemographics, HIV testing and sexual behavior, and access to health care. Questionnaire data were linked to laboratory data and result collection records to identify facilitators of result collection and preferred communication method.

Quantitative data were analyzed using SPSS 22 software (IBM). A first descriptive analysis was performed on all variables and stratified by gender. In the second univariate analysis, Pearson chi-square was used according to variable properties to compare proportions and identify factors associated with preference in result collection methods and actual result collection. A significance level of 5% was applied.

### Qualitative Methods

For an in-depth assessment of acceptability, facilitators, and barriers for participation, self-reported data were complemented with qualitative data from participant observations during 10 testing events. Two trained female social scientists, of Ugandan and Belgian origin, observed the 10 sessions and conducted informal interviews in English, French, or Dutch with 3 groups: testers, nontesters, and members of the intervention team. The researchers mingled with the crowd during the informational speech to observe people’s verbal and nonverbal reactions. Through this approach, people with particular viewpoints were identified and approached for an informal interview [29] striving for maximum variation in the sample. We adopted an unstructured interview approach [30], probing for the following themes: (1) perception and acceptability of outreach VCT in sub-Saharan African migrant community settings, (2) motivations for declining or accepting the test offer, (3) perceptions of and/or experiences with the oral fluid collection devices, (4) perception and acceptability of HIV test result collection via a secured website or a low threshold HIV and STI testing center. During participant observation and after informal interviews, the researchers took jottings, which were assembled into field notes the day after the session. Field notes were uploaded in N-VIVO 10, coded using a data-driven codebook established by 2 independent researchers. Following inductive analysis principles [31], emerging themes and their relationships were identified. Text segments that were coded differently were discussed until consensus was reached. A second-order analysis identified participant clusters based on gender, age, nationality, and type of setting.

### Ethical Considerations

The swab2know intervention was conceptualized and implemented in close collaboration with sub-Saharan African migrant community leaders. Ethical approval was obtained from the Institutional Review Board at the Institute of Tropical Medicine and the University Hospital in Antwerp.

## Results

### Quantitative Findings

#### Characteristics, Settings, and Participants

About 780 people were present in the selected venues and participated in the information session, and 142 (18.2%) underwent HIV testing. Table 1 summarizes participants’ characteristics and HIV test results.

#### HIV Testing Results and Behavioral Characteristics

The samples of 5 participants, all from men, were indicative of an HIV infection. About 29.8% of participants, more men than women, tested for the first time. An additional 44.1% reported that their last HIV test dated from more than 1 year ago, and 46.2% did not know their current HIV status, 22.3% had no GP, and 21.5% reported to have had 2 or more sexual partners during the last 3 months.

#### Result Collection and Linkage to Care

Of all participants, 56.3% chose to collect their results via the secured website, 43.7% opted for a consultation at the HIV and STI testing center. Participants who decided for Web-based result collection were more likely able to speak Dutch (*P*=.003) and came from settings with lower general acceptance of the intervention, as identified based on the qualitative data (see “Acceptability of Outreach HIV Testing in African Migrant Community Settings” section). We observed that at events and in bars, participants were more likely to opt for Web-based result collection, but this could not be confirmed statistically. The same was found for participants who already had tested before and those living abroad.

Overall, 78.9% of participants collected their HIV test result. Of those who opted for result collection at the testing facility, 85% collected their HIV test result, compared with 74% who chose the website. This difference was statistically not significant. Five participants who initially preferred to collect their results through the Internet came to the testing center because they failed to access the website. None of the associations with the variable result collection tested in the univariate analysis (as shown in Table 2) were statistically significant. Of the 5 participants with a reactive test result, 2 were confirmed HIV positive on blood, of which 1 was a new diagnosis and 1 was a patient already in medical follow-up. One test result was confirmed to be false positive on a blood sample. Two participants with a reactive result never showed up for confirmation testing. They were contacted by the study nurse and invited for counseling at the HIV and STI testing center. When they missed the first appointment, 8 additional attempts were made to contact them again, without success.

**Table 1:** Participants’ characteristics and HIV test results, stratified by gender.

		Total^a^	Male	Female
		N:142 (%)	N:73 (%)	N:60 (%)
Setting type				
	Church	32 (22.5%)	12 (16%)	15 (25%)
	Event	42 (29.6%)	29 (40%)	13 (22%)
	Bar	14 (9.9%)	12 (16%)	0 (0%)
	Information session	53 (37.3%)	20 (27%)	32 (53%)
Survey language				
	English	68 (47.9%)	29 (40%)	33 (55%)
	French	50 (35.2%)	33 (45%)	14 (23%)
	Dutch	24 (16.9%)	11 (22%)	13 (22%)
Age, years				
	Mean (rang)	38.5 (17-73)	38.2 (17-60)	38.6 (18-73)
	≤ 30	41 (29.3%)	20 (27%)	20 (33%)
	31-40	41 (29.3%)	27 (37%)	12 (20%)
	41-50	30 (21.4%)	11 (15%)	17 (28%)
	≥ 51	28 (20.0%)	15 (21%)	11 (18%)
Country of origin				
	Central Africa (DRC, Angola, Cameroon)	61 (43.0%)	40 (55%)	17 (28%)
	East Africa (Burundi, Kenya, Somalia, Tanzania, Uganda, Rwanda, and Zambia)	10 (7.0%)	4 (5%)	6 (10%)
	West Africa (Ivory coast, Ghana, Mali, Liberia, Nigeria, Senegal, Cape Verde)	51 (35.9%)	24 (33%)	22 (37%)
	Southern Africa (South Africa)	2 (1.4%)	0 (0%)	2 (3%)
	European (Belgium and France)	8 (5.6%)	3 (4%)	5(8%)
	Other (Curaçao, Jamaica, Suriname, Guyana, and Morocco)	10 (7.0%)	2 (3%)	8 (13%)
Country of residence				
	Belgium	135 (95.1%)	70 (96%)	56 (93%)
	Other (Netherlands, UK, France, Sweden, and Canada)	7 (4.9%)	3 (4%)	4 (7%)
Access to primary care^b^				
	Has a general practitioner (GP)	108 (77.7%)	53 (76%)	48 (80%)
	Does not have a GP	31 (22.3%)	17 (24%)	12 (20%)
Sexual preference^c^				
	Heterosexual	109 (93.2%)	59 (92%)	50 (94%)
	Gay, Lesbian, or bisexual	8 (6.8%)	5 (8%)	3 (6%)
Number of sexual partners in last 3 months^d^				
	None	29 (21.5%)	12 (17%)	13 (22%)
	1	77 (57.0%)	40 (57%)	35 (59%)
	2-6	29 (21.5%)	18 (26%)	11 (19%)
Last HIV test^e^				
	Less than 1 year ago	35(26.1%)	21 (31%)	12 (21%)
	Between 1 and 3 years ago	36 (26.9%)	15 (22%)	19 (33%)
	More than 3 years ago	23 (17.2%)	10 (15%)	12 (21%)
	Never tested	40 (29.8%)	22 (32%)	14 (25%)
Reported believed HIV status (questionnaire data)^f^				
	HIV-negative	70 (53.8%)	34 (52%)	33 (57%)
	Unknown	60 (46.2%)	32 (48%)	25 (43%)
Result oral fluid sample (laboratory data)				
	HIV-negative	137 (96.5%)	68 (93%)	60 (100%)
	Reactive	5 (3.5%)	5 (7%)	0 (0%)

^a^For 9 participants, gender data are missing.

^b^Data missing of 3 participants.

^c^Data missing of 25 participants.

^d^Data missing of 7 participants.

^e^Data missing of 8 participants.

^f^Data missing of 12 participants.

**Table 2:** Comparison of participant and intervention characteristics for preferred HIV test result collection method and result collection (N=142).

		Collection method			HIV test result collected		
		Testing center	Secured website	*P* value	Yes	No	*P* value
		N:62 (43.7%)	N:80 (56.3%)		N:112 (78.9%)	N:30 (21.1%)	
Setting type							
	Church	16 (50%)	16 (50%)	.06	28 (87.5%)	4 (12.5%)	.20
	Event or bar	18 (32%)	39 (68%)		41 (72%)	16 (28%)	
	Information session	28 (53%)	25 (47%)		43 (81%)	10 (19%)	
Acceptability in setting^a^							
	High acceptance	42 (52%)	39 (48%)	.02^c^	67 (83%)	14 (17%)	.20
	Low acceptance	20 (33%)	41 (67%)		45 (74%)	16 (26%)	
Survey language							
	English	34 (50%)	34 (50%)	.003^c^	56 (82%)	12 (18%)	.26
	French	25 (50%)	25 (50%)		40 (80%)	10 (20%)	
	Dutch	3 (12.5%)	21 (87.5%)		16 (67%)	8 (33%)	
Result collection method							
	HIV and STI testing center			^d^	53 (85%)	9 (15%)	.09
	Secured website				59 (74%)	21 (26%)	
Gender^b^							
	Male	37 (51%)	36 (49%)	.10	56 (77%)	17 (23%)	.65
	Female	22 (37%)	38 (63%)		48 (80%)	12 (20%)	
Age^b^, years							
	≤ 30	13 (32%)	28 (68%)	.17	27 (66%)	14 (34%)	.06
	31-40	23 (56%)	18 (44%)		37 (90%)	4 (10%)	
	41-50	13 (43%)	17 (57%)		24 (80%)	6 (20%)	
	≥ 51	13 (46%)	15 (54%)		22 (79%)	6 (21%)	
Country of origin							
	Central Africa	25 (41%)	36 (59%)	^d^	45 (74%)	16 (26%)	^d^
	East Africa	4 (40%)	6 (60%)		7 (70%)	3 (30%)	
	West Africa	27 (53%)	24 (47%)		43 (84%)	8 (16%)	
	Southern Africa	0 (0%)	2 (100%)		2 (100%)	0 (0%)	
	Belgium and France	3 (37.5%)	5 (62.5%)		7 (87.5%)	1 (12.5%)	
	Other	3 (20%)	7 (70%)		8 (80%)	2 (20%)	
Residence							
	Belgium	62 (45.9%)	73 (54.1%)	^d^	106 (78.5%)	29 (21.5%)	^d^
	Abroad	0 (0%)	7 (100%)		6 (86%)	1 (14%)	
General practitioner^b^							
	No	13 (42%)	18 (58%)	.88	25 (81%)	6 (19%)	.82
	Yes	47 (43.5%)	61 (56.5%)		85 (78.7%)	23 (21.3%)	
Sexual Preference^b^							
	Heterosexual	50 (46.7%)	57 (53.3%)	^d^	85 (80.1%)	21 (19.8%)	^d^
	Gay, Lesbian, or bisexual	5 (62.5%)	3 (37.5%)		5 (62.5%)	3 (37.5%)	
Number of partners^b^							
	None	14 (48%)	15 (52%)	.64	20 (69%)	9 (31%)	.08
	1	33 (43%)	43 (57%)		60 (79%)	16 (21%)	
	2-6	15 (54%)	13 (46%)		26 (93%)	2 (7%)	
Testing history							
	Never tested	22 (55%)	18 (45%)	.08	31 (77.5%)	9 (22.5%)	.82
	Ever tested	39 (38.6%)	62 (61.4%)		80 (79.2%)	21 (20.8%)	
Reported HIV status^b^							
	HIV negative	26 (37%)	44 (63%)	.27	58 (83%)	12 (17%)	.27
	Unknown	28 (47%)	32 (53%)		45 (75%)	15 (25%)	
Result oral fluid sample (laboratory data)							
	Negative	60 (46.8%)	77 (53.2%)	^d^	109 (79.6%)	28 (20.4%)	^d^
	Reactive HIV test	2 (40%)	3 (60%)		3 (60%)	2 (40%)	

^a^Categorization based on the qualitative data (see “Acceptability of Outreach HIV Testing in African Migrant Community Settings” paragraph).

^b^Cases with missing data have been excluded from the univariate analysis.

^c^Significant difference at 5% level.

^d^ Indicates that *P* value could not be calculated because of the limited number of cases in 1 of the variables.

### Qualitative Findings

We collected a total of 137 field notes, 41 of informal interviews from testers who tested for HIV, 53 of nontesters, and 16 of people who were still undecided. In addition, 14 conversations were held with the intervention team, and 13 descriptive notes of observations were collected.

#### Acceptability of Outreach HIV Testing in African Migrant Community Settings

Based on the qualitative data, settings with high versus low acceptance for outreach HIV testing interventions emerged. Acceptance referred to people’s attitudes toward the intervention and its methods, that is, participants expressed appreciation, which did not necessarily translate into participation. In settings frequented by women and adults aged between 30 and 50 years, for example, churches and women’s organizations, acceptance was rather high. Other factors related to acceptance clearly emerging from the data were community ownership, prior HIV awareness, and the perceived appropriateness to test in such settings. Moreover, communities familiar with HIV prevention campaigns, such as the Congolese and the LGBT communities, and also those whose when leaders had sensitized their members in advance, accepted the intervention more easily than others. At the World AIDS day event, a man aged 40 to 50 years old told me:

This event is a good day to test. It is a mixture of celebrations to remember the people who have passed away and those still living with HIV.

At a Nigerian cultural event, a man in his forties told me he could not understand why this special event had to be interrupted by an activity that has nothing to do with the festival (...) “The focus should be on the king and nothing else.

The aforementioned quotes demonstrate the close link between general acceptance and the perceived appropriateness. Mixing HIV with cultural celebrations and parties was disapproved of, particularly when leaders failed to openly take ownership for the testing offer. Settings, where statements like *“Go to the people who need it”* were commonly heard, for instance, calling on *“prostitutes and womanizers in need of HIV-testing,”* were subsequently categorized as settings with lower acceptance. In such cases, members of the audience often did not perceive themselves as being at risk of HIV and therefore accepted outreach HIV testing to a lesser extent. Regardless of the confidential procedure, participants seemed to fear social control, due to closely knitted social networks: At the information evening of the Ghanaian association, a lady in her thirties told me:

Someone can ask: “Oh, last time you went for testing. How was it?” Then you cannot tell lies all the time, you will have to tell them. If you went to the doctor privately, you can choose the time to share such news.

In settings with lower acceptance, however, prevention benefits seemed to be larger. This finding is supported through the laboratory data, as the 5 reactive cases were all found in the settings with lower acceptance. Many people at these settings also indicated that the information session and in particular, the testimony of a community member living with HIV had raised their awareness of HIV and changed their views about HIV testing. They often mentioned their intention to test later, for instance at their GP or asked concrete information about the HIV and STI testing center.

At the Nigerian cultural event, I approached a lady in her thirties. When I asked her thoughts about the testing offer, she answers: “Take that message to the people who need it.” (...) When the witness came back to her seat [after her testimony], I saw that she asked her for her business card (...) When I approached her again, she explained she would not test at that particular day but when she reaches home she would read more information on the website.

#### Facilitators and Barriers for Uptake of HIV-Testing

Participants expressed their motivation for testing often through statements like *“it is good to know your status.”* Participants said the team’s presence reminded them of the need to test and they felt encouraged by the informational speech and testimony. Participants also wanted to benefit of the opportunity to get a free test without having to visit a physician.

At a church, a man in his forties told me that they [the swab2know team] explained it well and it is a good occasion to test and know it. If you go for checkups, like headache or something else, there is no time for a test. He thinks it’s good that he doesn’t have to go to the hospital and waste more time there.

Only few participants referred to their previous testing behavior: either they never tested before, tested regularly, or they wanted to confirm previous test results. In some cases, peer and partner pressure were also at play:

At church, a woman in her thirties told me she was not going to test because she had given birth to all of her children in Belgium and “surely, if there was something wrong the doctors would have said it. (...) A bit later the pastor’s wife joined our conversation and told her she was going to have a test. The first lady changed her mind and went along. Later she told me: “it feels good to test, it was good and simple.”

As this quote illustrates, people who declined referred to a previous test. However, probing showed that Africans often assumed they had been tested. Some participants believe that an HIV test is standard procedure with every blood analysis carried out, and because *“the doctor never mentioned anything,”* they were convinced to be HIV negative. Men also believed frequently to be HIV negative by association:

At the Cameroonian cultural event, a man in his thirties told me he sees no reason to test. (...) All his previous girlfriends have gone to marry and give birth.

Others declined the testing offer because they assessed their personal risk to be low, for instance, women, who were married or reported to use condoms consistently. Men made risk assessments of their partners:

Arriving at the Nigerian event, I started talking to a young boy aged 20-30 who is the photographer. He tells me that he has a “non-serious girlfriend” (...) When I ask him what he thinks of the testing offer he says: “I am only with my girlfriend, I don’t have to test.” When I ask him if he knows her HIV status, he says: “he virginized her” (...) “I am not a womanizer. There are lots of womanizers. Also I, myself, I can get a lot of women with this business, the girls come and show off in front of my camera (...) “No, I never tested, but I am sure I don’t have it. I trust my girl.”

#### Perception of Oral Fluid Collection Devices

Almost all testers said that the oral fluid collection devices were easy to use and painless. They welcomed them as a good alternative for blood samples:

The Ghanaian pastor in his forties told me that coming to church he was thinking about how they were going to take the blood and “drain us.” He thought they were going to take three bottles, a small and two big ones and in the end the result would be the same. He was happy with the “saliva” test, he said “new technology is good, because people don’t like blood taking.”

Clearly, the use of oral fluid collection devices had convinced many participants to test and as such lowered their thresholds for HIV testing. However, the method also raised misconceptions pertaining to HIV transmission modes, which needed to be addressed by the intervention team:

At the Nigerian cultural event a women in her thirties told me that she never would have guessed that now HIV can be found in saliva. “What’s next? Are we now going to find it in sweat or if we shake hands?” I told her that she can ask the doctor but HIV could not be transmitted through handshakes. She answered “My sister, don’t be too sure, it is also liquid like saliva.”

#### Web-Based Result Collection Versus Counseling at the HIV or STI Testing Center

The informal interviews also focused on the result collection method chosen and participants’ motives for their decision. Those who selected the website appreciated the convenience of not having to comply with the opening hours of the HIV and STI testing center. Some added *“the website is okay, because I do not have anything.”* Others said it was their incentive to test because it enabled participation of sub-Saharan African migrants living outside the Antwerp region.

Participants who chose for result collection via the testing center mostly referred to technical barriers in relation to the website, such as no Internet access, no email address, or low Internet skills. Interestingly, only few expressed the need for professional counseling:

At church, a man in his twenties told me that he wants his results from Helpcenter-ITG because “with the website you might not be able to understand it well, there might be miscommunication. It is good that they sit you down and explain to you what you have to do.”

## Discussion

This study adopted mixed methods to assess the acceptability of outreach VCT, using oral fluid collection devices and offering Web-based result collection among a sample of sub-Saharan African migrants. Our findings show that when accompanied by tailored provision of information, these innovative methods are acceptable and are perceived to reduce thresholds for HIV testing uptake. Using a community-based participatory settings approach, we were able to reach migrants from high endemic regions, that is, 87.3% originated from sub-Saharan Africa. Almost 1 in 5 participants (ie, 18.2%) present at the respective settings participated. This is considerably more than in a previous intervention undertaken in comparable community settings, where only 8.7% of the people present gave a blood sample for HIV testing, and results were only available from the HIV and STI testing center [21]. The qualitative data showed that especially sampling via oral fluid collection devices reduced barriers to uptake because sub-Saharan African migrants dislike blood taking. Offering the choice between 2 result collection methods—via a secured website or STI or HIV testing facility—attracted participants with different needs. Although only 1 participant was successfully linked to HIV care, the intervention shows some potential to reach subgroups at increased risk for HIV and with reduced access to mainstream testing opportunities. Overall, 29.8% reported testing for HIV for the first time, 22.3% did not have a GP, and 21.5% reported 2 or more sexual partners in the last 3 months.

The mixed-method research approach of quantitative and ethnographic methods to assess acceptability resulted in in-depth knowledge of the facilitating and hindering factors, describing the complexity of the decision-making process for testing uptake. Uptake was highly dependent of leaders’ ownership and perceived appropriateness of the event, as shown in similar initiatives [32]. Acceptance was highest in churches, underlining their potential for future outreach testing. Similar to others, we identified that HIV test result collection was better in church settings [33], confirming the influential role of religious leaders in HIV stigma reduction and HIV testing uptake. Although overall, more men than women participated—which can be explained by the male dominance in community settings—the qualitative data revealed that men were less accepting of the testing initiative and more reluctant to test. This is in line with UK research [34,35]. Motivating their refusal, especially men seem to make risk assessments based on assumptions of their current and previous partners and themselves. In addition, misconceptions about how HIV tests would be regularly performed by health care providers and transferability of the (ex-) partners’ test result were still prevailing. These qualitative findings have implications for HIV prevention, calling for a gender-specific approach and for addressing culturally grounded misconceptions. Comparable to similar research [36], we identified that sub-Saharan African migrants with poor protective behavior reported low perceived risk. This stresses the importance of qualitative and tailored provision of information accompanying outreach HIV testing.

Most testers in our study were opportunistic testers [26], motivated by their preexisting attitudes toward health and HIV testing. Those already convinced of the importance of regular testing perceived the noninvasive oral fluid collection devices as an additional incentive. Testimony given by community members living with HIV was able to motivate initially skeptical participants, which underlines the importance of involving people living with HIV in HIV prevention [26,37]. However, the qualitative data also demonstrated the effect of peer and partner pressure on decision taking, which may be typical for close-knitted communities. Compared with another outreach HIV testing project with delayed result communication among African Americans (46% among uninfected and 66% among infected participants; [25]), result collection was high (79%) in our study. Yet, from the perspective of linkage to care, the following considerations need to be made. Participants who opted for the website delivery of the HIV test results more often failed to pick up their results than participants who chose delivery at the testing center. Qualitative data identified lack of technical skills and access to the Internet as main barriers, indicating that communicating results only through a secured website are not yet advisable for this target group. Regardless of our efforts to assure linkage to care, we failed to follow-up 2 of 5 persons with a reactive test results. As late diagnosis and delayed presentation to care after HIV diagnosis are more common in the subgroup of sub-Saharan African migrants [38], decreasing anonymity in favor of collecting more person-identifying data should be debated. To increase participation, we opted to fully anonymize data. However, the qualitative data showed that the spatial limitation of certain settings, closely knitted social networks, and influence of social desirability and leaders undermine these efforts. In practice, anonymous participation can neither be fully guaranteed nor could it be identified as a decisive factor for HIV test uptake. Rather, confidentiality and reliability of the test results are priorities for sub-Saharan African migrants [39]. Indicating that collecting more person-identifying data to improve linkage to care would be acceptable.

This study has some methodological limitations: We relied partly on self-reported data; thus, participants may have given social desirable answers. Study participants were self-selected, which potentially introduced a bias. The qualitative data collection was carried out among a subsample of all intervention participants, and although we have tried to maximize their variation, this may also have influenced the outcome. Due to the practical constraints at the settings, we could not afford to ask detailed questions in the questionnaire. For instance, questionnaire data on sexual behavior did not include past and current preventive behaviors such as condom use. Finally, the study was limited to 10 outreach HIV testing sessions and included a small convenience sample; therefore, results cannot be generalized. Notwithstanding these limitations, the findings generate interesting implications for future community-based outreach HIV testing interventions among migrants.

### Conclusion

This mixed-method evaluation of the “Swab2know” intervention targeting sub-Saharan African migrants yielded promising results. Such interventions need to be replicated and evaluated more rigorously before upscaling. The intervention was able to reach sub-Saharan African migrants and other migrants from high endemic regions at higher risk of HIV infection and with limited access to regular HIV testing: almost, a third tested for the first time, 21.5% reported having had 2 or more sexual partners in the last 3 months, and 22.3% reported not having a GP. The qualitative data demonstrated the additional prevention benefit of outreach VCT. Prevention outcome was high especially in settings with lower acceptance: for many participants, the presence of the intervention team visualized the magnitude of the HIV epidemic and promotes HIV testing uptake at large. The fact that many people indicated they would postpone testing to a later moment, for instance, with their GP, calls for a multipronged HIV test promotion strategy targeting communities and health care providers alike [40] to increase HIV testing uptake among vulnerable and diverse migrant communities.

## Abbreviations

GP: general practitioner
HIV: human immunodeficiency virus
LGBT: lesbian Gay Bisexual Transsexuals
MSM: men who have sex with men
STI: Sexually Transmitted Infections
VCT: voluntary HIV counseling and testing
